# Cool Farm Tool Water: A global on-line tool to assess water use in crop production

**DOI:** 10.1016/j.jclepro.2018.09.160

**Published:** 2019-01-10

**Authors:** Benjamin Kayatz, Gabriele Baroni, Jon Hillier, Stefan Lüdtke, Richard Heathcote, Daniella Malin, Carl van Tonder, Benjamin Kuster, Dirk Freese, Reinhard Hüttl, Martin Wattenbach

**Affiliations:** a GFZ German Research Centre for Geosciences, Telegrafenberg, 14473, Potsdam, Germany; b Institute of Biological & Environmental Sciences, University of Aberdeen, 23 St Machar Drive, Aberdeen, AB24 3UU, UK; c Department Computational Hydrosystems, Helmholtz Centre for Environmental Research – UFZ, Permoserstrasse 15, 04318, Leipzig, Germany; d Institute of Earth and Environmental Sciences, University of Potsdam, Karl-Liebknecht-Strasse 24–25, 14476, Potsdam, Germany; e Global Academy of Agriculture and Food Security, The Royal (Dick) School of Veterinary Studies and the Roslin Institute, Easter Bush Campus, Midlothian, EH25 9RG, UK; f Cool Farm Alliance, The Stable Yard, Vicarage Road, Stony Stratford, MK11 1BN, UK; g Anthesis, 9 Newtec Place, Magdalen Road, Oxford, OX4 1RE, UK; h Virtual City Systems, Tauentzienstrasse 7 b/c, 10789, Berlin, Germany; i Brandenburg Technical University Cottbus-Senftenberg, Platz der Deutschen Einheit 1, 03046, Cottbus, Germany

**Keywords:** Water footprint, FAO56, Crop water use, Stakeholder involvement, Water resource management, Irrigation requirements

## Abstract

The agricultural sector accounts for 70% of all water consumption and poses great pressure on ground water resources. Therefore, evaluating agricultural water consumption is highly important as it allows supply chain actors to identify practices which are associated with unsustainable water use, which risk depleting current water resources and impacting future production. However, these assessments are often not feasible for crop producers as data, models and experiments are required in order to conduct them. This work introduces a new on-line agricultural water use assessment tool that provides the water footprint and irrigation requirements at field scale based on an enhanced FAO56 approach combined with a global climate, crop and soil databases. This has been included in the Cool Farm Tool – an online tool which already provides metrics for greenhouse gas emissions and biodiversity impacts and therefore allows for a more holistic assessment of environmental sustainability in farming and agricultural supply chains. The model is tested against field scale and state level water footprint data providing good results. The tool provides a practical, reliable way to assess agricultural water use, and offers a means to engage growers and stakeholders in identifying efficient water management practices.

## Highlights

• CFTW facilitates water use assessments in crop production in food supply chains.
• Global on-line tool combining user input with a soil, daily climate and crop database.
• Comparison to observed evapo-transpiration ($R^{2}$  = 0.52) shows good agreement.
• Comparison to observed total water footprint ($R^{2}$  = 0.96) shows very good agreement.
• Using CFTW can reduce errors in water footprints by >70% compared to state estimates.

## 1 Introduction

With increasing global food demand, agricultural water use and consequent ground water depletion, improved farm water management is becoming increasingly critical (Godfray et al., 2010; Siebert et al., 2010; Tilman et al., 2011; Wada et al., 2012). A global modelling study by Jägermeyr et al. (2016) investigated different integrated crop water management interventions, including an increase of irrigated areas. The study indicates that production could be increased by 41% and thus the gap in future global food demand could be reduced by 50% - but not without further increasing irrigation water consumption. Therefore a solid understanding and estimation of crop water usage, crop water demand and the effect of different water management at farm level is crucial to enable the identification of improved management opportunities.

Several models, of varying complexity, have been developed in order to account for water use in crop production at the field scale (Baroni et al., 2010; Kroes et al., 2008; Raes et al., 2006; Ragab, 2002; Rosa et al., 2012; Smith, 1992; Steduto et al., 2009). Most of them use, to some extent, the approach presented in the “FAO irrigation and drainage paper No. 56 crop evapotranspiration” (FAO56) (Allen et al., 1998). However, these models are often not tailored to application by crop producers, due to (i), the unavailability of soil, crop and climate data required for the model, (ii), the use of terminology not understood outside the research community, (iii), lack of an engaging user interface for some models, in addition to (iv), a lack of guidance on how to interpret and use results. Bastiaanssen et al. (2007) raised similar concerns for soil hydrological models. Table 1 gives a short overview of some of the existing tools based on FAO56. The selection is based on models described in the scientific literature and the provision of a graphical user interface.

**Table 1:** Overview of existing field water assessment tools that deploy the FAO56 approach (Allen et al., 1998). The table provides the level of data integration for climate, soil and crop. Most tools allow the users to update existing soil and crop information.

Name	Source	Climate data	Crop parameters	Soil parameters	Special features
AquaCrop	FAO, Steduto et al. (2009)	database with 5000 stations (CLIMWAT)	14 default crops	14 default soil profiles	contains a full crop growth model for yield prediction including different stresses
CRIWAR	Bos et al. (2008)		10 default crops	only needed when determining water requirements	
CROPWAT	FAO, Smith (1992)	database with 5000 stations (CLIMWAT)	36 default crops	3 default soils	
ICARDA Agro-Climate tool	Mauget and De Pauw (2010)	interpolation between 649 climate stations	default crops provided	default soil types provided	only applicable for north-west Africa to central Asia
MABIA-Region	Allani et al. (2012)		>100 default crops	12 default soil texture classes	GIS based
SALTMED	Ragab (2002)		>200 default crops	40 default soils	includes advanced soil water model & use of saline water for irrigation
SAPWAT	van Heerden (2008)	database with 5000 stations (CLIMWAT) & South African climate station data	default crops provided	default soils provided	climate station data available for South Africa
SIMDualKc	Rosa et al. (2012)		auxiliary data provided	auxiliary data provided	
SPARE-WATER	Multsch et al. (2013)				GIS based

The models vary with respect to data integration, with most data being provided by the ICARDA Agro-Climate tool (Mauget and De Pauw, 2010) and SAPWAT (van Heerden, 2008) for north-west Africa to central Asia and South Africa, respectively. CROPWAT, SAPWAT and Aquacrop provide climate data on a global scale via the climate database CLIMWAT, which contains long-term average data from 5000 climate stations (van Heerden, 2008; Smith, 1992; Steduto et al., 2009). The data can also be downloaded and used for the other existing models. Most tools provide default soil profiles and parameters, but do not use soil maps to increase usability.

This study presents the new field scale agricultural water assessment tool Cool Farm Tool Water (CFTW) which is fully integrated with the already existing greenhouse and biodiversity model Cool Farm Tool (CFT) (Hillier et al., 2011). The novelty of this tool is that it combines tested algorithms with a database of climate, soil and crop data on a global scale in an on-line tool and packages them for non-expert use with limited data availability. In doing so, some of the above documented shortcomings of existing models are improved. With CFTW, agricultural water assessments can now be performed using local information on production, climate and management. Growers, companies and non-governmental organisations are thus no longer dependent on national or regional datasets, own modelling or measurement work to assess their water use. CFTW provides results on the water footprint (WFP), which describes the water consumed per unit product as well as irrigation requirements. Furthermore, it provides the possibility to compare different production sites and systems using the same methodology. Finally, together with the already existing on-line tool CFT, it enables crop producers and stakeholders to take a more informed and holistic approach on environmental sustainability in the agricultural sector.

In this study we first introduce the existing CFT as the foundation of CFTW (section 2). CFTW is then presented in detail, describing the model, the database, and the user interface (section 3). To understand the effect on the accuracy of using global datasets for determining WFPs, the tool is evaluated based on 16 studies available in the literature in different climatic and soil-plant conditions (section 4). The study provides also one of the first assessments of different modelled WFPs with observations. Finally, limitations and future developments are discussed and concluding remarks presented.

## 2 Cool Farm Tool - CFT

The development of the CFT (https://coolfarmtool.org) started in 2008 as an on-farm greenhouse gas (GHG) emission calculator based on a collaboration between the University of Aberdeen, the Sustainable Food Lab and Unilever. The GHG tool captures emissions related to crop and livestock production. Emissions are determined using empirical models and emission factors which consider differences between production systems, regions and climates (Aryal et al., 2015; Hillier et al., 2011). The interest in the tool from consumer good producers, retailers, non-governmental organisations, fertilizer producers and small and medium-sized enterprises led to the formation of the Cool Farm Alliance (CFA) in 2014, which now manages and owns the tool. The CFA currently has over 53 members who are using and co-developing CFT in collaboration with academics across several research organisations.

The tool was first developed as an Excel spreadsheet and published in 2011 (Hillier et al., 2011). In 2012, CFT on-line was released and has been used by 4900 registered users. Usage requires a one time registration on https://coolfarmtool.org and enables the user to assess up to five crops.

The tool has also been applied in over 30 scientific publications over the last 6 years. The scope of the different studies ranged from model comparisons (Camargo et al., 2013; Colomb et al., 2013), to product assessments of, for example wheat, potato and coffee (Aryal et al., 2015; Haverkort et al., 2014; Sapkota et al., 2014) as well as investigations of mitigation strategies at the global scale (Hillier et al., 2012).

Based on further requests by the different members of the CFA, the tool was extended with the biodiversity module and the water module. The biodiversity module was released in 2016 and is based on the Gaia biodiversity yardstick (CFA, 2016; CLM, 2017). It provides an evidence-based biodiversity assessment for the north-west European biome. The water module has been released in 2017 and is described and assessed in the present study.

## 3 Cool Farm Tool Water - CFTW

**Fig. 1:**
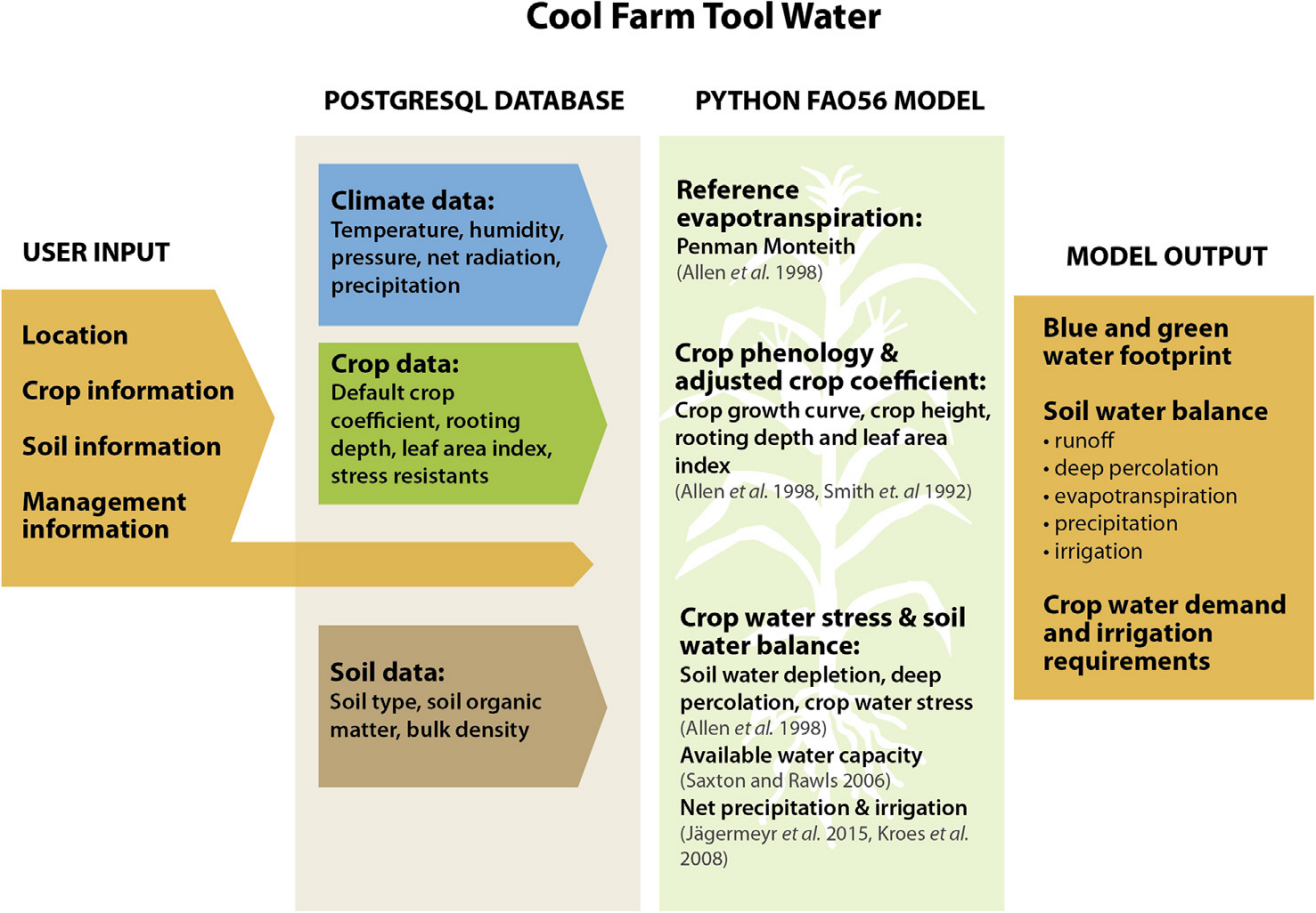
Schematic representation of CFTW model components and related publications. The figure also shows where CFTW makes adjustments to FAO56, by introducing different or new model components. A more detailed visual description of the model is presented in the Appendix B.

The CFTW is programmed in Python 2.7. It estimates crop water use and the main components of the soil water balance combining the single crop coefficient approach presented in the “FAO irrigation and drainage paper No. 56 crop evapotranspiration” (Allen et al., 1998) with global datasets for soil, crops and climate. Adjustments to crop phenology, soil water balance simulations and management options have been made to increase accuracy, represent current knowledge or to enhance usability. The adjustments are described in the following section 3.1 and summarised in Fig. 1. Finally, model and data are integrated on-line and accessed via a user-friendly interface at https://coolfarmtool.org using any internet browser.

### 3.1 Model

#### 3.1.1 Actual evapotranspiration $ET_{a}$

The single crop coefficient approach and - thus CFTW - determines actual evapotranspiration $ET_{a}$ (mm d^−1^) based on three distinct steps (Allen et al., 1998). First, the reference evapotranspiration $ET_{0}$ (mm d^−1^) is estimated based on the Penman-Monteith equation. $ET_{0}$ refers to a short well-watered grass with an assumed crop height of 0.12 m, a fixed surface resistance of 70 s m^−1^ and an albedo of 0.23 (Allen et al., 1998). Based on these values, $ET_{0}$ is determined as follows:

(1) $$ET_{0}=\frac{0.408\cdot \text{Δ}\cdot \left(R_{n}-G\right)+\gamma \cdot \frac{900}{T+273}\cdot u_{2}\cdot \left(VPD\right)}{\text{Δ}+\gamma \cdot \left(1+0.34\cdot u_{2}\right)}$$

where $R_{n}$ is the net radiation at the crop surface (M J m^−2^ d^−1^), *G* is soil heat flux density approximated as 0 M J m^−2^ d^−1^ on a daily basis, *T* and $u_{2}$ are the air temperature (^°^C) and wind speed (m s^−1^) at 2 m height, VPD is the vapour pressure deficit of the air (kPa), *γ* is the psychometric constant (k Pa ^°^C^−1^), *λ* is the latent heat of vaporization (MJ kg^−1^) and Δ is the slope of the saturation vapour pressure vs. air temperature curve (kPa ^°^C ^−1^). In the second step, $ET_{0}$ is corrected based on the single crop coefficient $K_{c}$ to determine the potential crop evapotranspiration $ET_{c}$ (mm d^−1^) as follows:

(2) $$ET_{c}=ET_{0}\cdot K_{c}$$

**Fig. 2:**
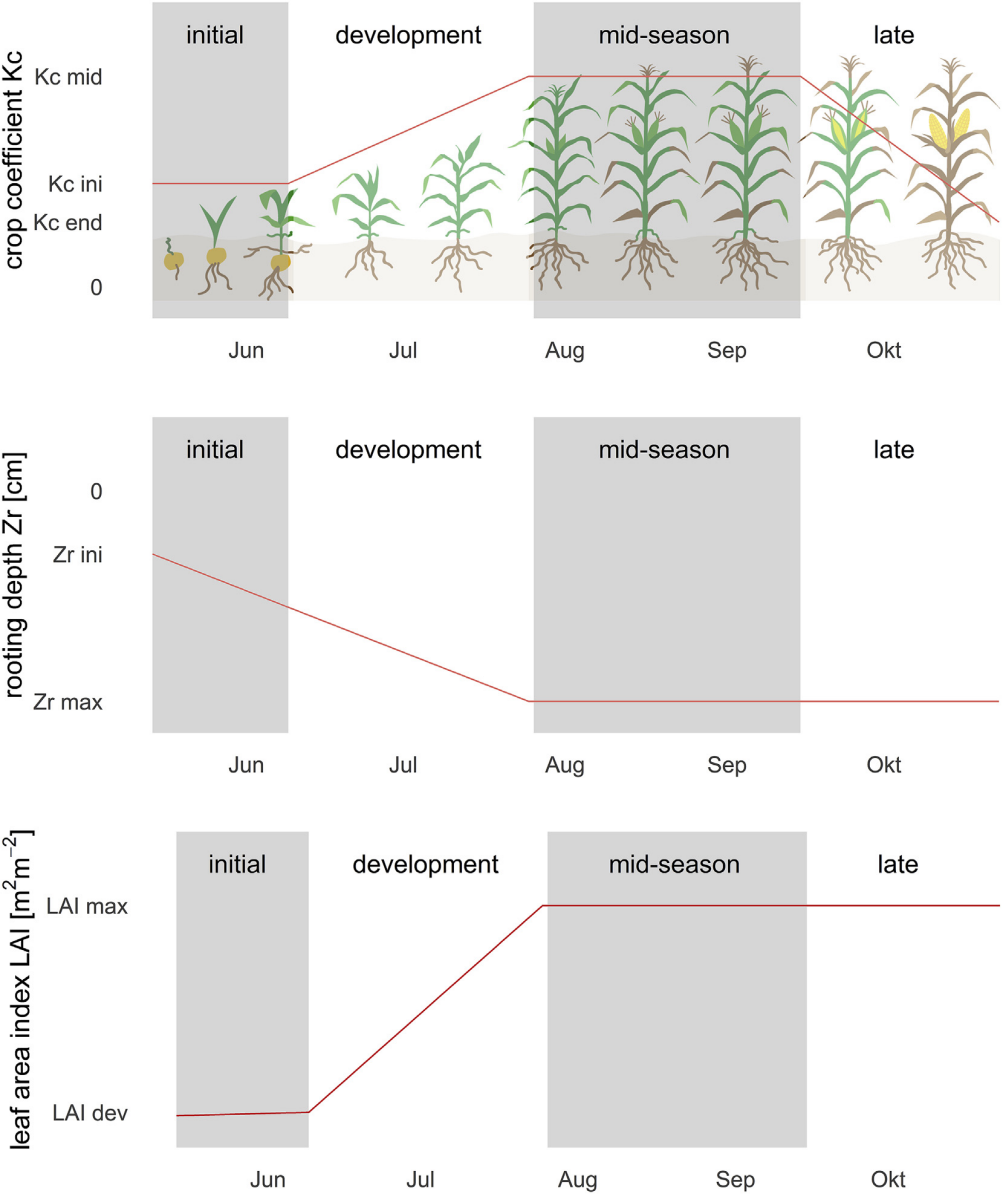
The schematic plot shows the crop phenology in CFTW as represented by the crop growth curve showing the crop coefficient $K_{c}$, rooting depth $Z_{r}$ and the leaf area index LAI.

The single crop coefficient $K_{c}$ combines evaporation and crop transpiration into a single coefficient and scales the $ET_{0}$ so that it resembles a specific crop without any limitation of water and nutrients (Allen et al., 1998). The $K_{c}$ is not constant over the growing season as shown in the crop growth curve in Fig. 2.

$K_{c}$ is based on adjusted empirical values for various crops and linear interpolation between an initial, mid-season and end $K_{c}$ over the different crop growing periods. The literature values of $K_{c}$ are corrected to account for local climate, crop, soil and irrigation management conditions based on the approaches presented in Allen et al. (1998). $ET_{c}$ at the beginning of the growing period is primarily governed by evaporation from the top soil. Therefore, $K_{c}$ for the initial phase is defined by the wetting frequency of the soil surface, ET_0_, soil texture and the irrigation method. The remaining mid-season and late growing period are mostly dependent on crop type and are corrected for humidity and crop height. CFTW does not correct for wind speed as this is greatly influenced by field location and its surroundings and uses a global average of 2 m s^−1^ as recommended by FAO56 (Allen et al., 1998). Even though global wind speed data is available, they are often not representative at local scale. Finally, the length of the different growing periods are crop specific and scaled to the length of the total growing period defined by the user.

In the last step, $ET_{c}$ is scaled based on a water stress coefficient $K_{s}$ that accounts for the soil water available for transpiration for the plant and for evaporation and limits $ET_{c}$ to actual evapotranspiration $ET_{a}$ (mm d^−1^):

(3) $$ET_{a}=ET_{c}\cdot K_{s}$$

$K_{s}$ ranges between 0 and 1 and is defined by root zone depletion $D_{r}$, which is the water lost from the total available water to the plant and described in section 3.1.2.

(4) $$\begin{array}{r} K_{s}=\frac{TAW-D_{r}}{\left(TAW-RAW\right)}\;\text{for}\;D_{r}>RAW\\ K_{s}=1\;\text{for}\;D_{r}\leq RAW \end{array}$$

where TAW and RAW are the total and readily available water (mm) respectively. TAW represents the total storage capacity ($\theta_{FC}$ - $\theta_{WP}$) $Z_{r}$, where $\theta_{FC}$ and $\theta_{WP}$ are soil moisture at field capacity and at permanent wilting point, respectively and $Z_{r}$ is the rooting depth. RAW represents the part of TAW for which plants do not suffer water stress. In contrast to FAO56, where $Z_{r}$ is described as constant, $Z_{r}$ grows from an initial depth to the maximum depth over the initial and developing growth stage in CFTW (Fig. 2). This is an important adjustment also made by CROPWAT for example as not all soil water within the maximum rooting zone is available to the plant from the beginning of the growing period and neglecting this may lead to an underestimation of crop water stress (Bos et al., 2008).

#### 3.1.2 Soil water balance

The soil water balance, as expressed in terms of soil water depletion in the root zone $D_{r}$ at time *i*, is defined by a traditional tipping bucket approach (Allen et al., 1998). The bucket size is defined by field capacity and permanent wilting point described by the pedo-transfer function in Saxton and Rawls (2006) as well as the maximum $Z_{r}$ of the specific crop. The water balance can be written as follows:

(5) $$D_{r,i}=D_{r,i-1}-{\left(P+I-RO-Int_{I}\right)}_{i}+ET_{a,i}+DP_{i}-CR_{i}\pm LF_{i}$$

where *P* is precipitation, *I* is applied irrigation depth, CR is capillary rise, LF is the lateral soil water fluxes, RO is runoff from the soil surface from irrigation and precipitation, $Int_{I}$ is the interception loss from irrigation and DP is water loss out of the root zone by deep percolation. All the components are expressed in term of time step day *i* in mm d^−1^.

Initial soil water depletion is provided by the user and then simulated daily using the daily water balance. As in Allen et al. (1998) and Bos et al. (2008), CFTW assumes that LF and CR are negligible and, for this reason, not simulated. Therefore, CFTW is currently only applicable when these terms are small and do not influence the soil water balance significantly. Precipitation and irrigation are provided by a global data base and users, respectively. Net soil water infiltration is defined by net precipitation and net irrigation, which considers interception loss, surface run-off, and deep percolation. $Int_{I}$ is based on Hoyningen-Huene (1983) and Braden (1985) (Kroes et al., 2008).

(6) $$Int_{P+I,i}=a*LAI_{i}*\left(1-\frac{1}{1+\frac{b*P_{tot,i}}{a*LAI_{i}}}\right)$$

where $Int_{P+I}$ (mm d^−1^) is intercepted precipitation and irrigation on day *i*, *a* is an empirical coefficient of 0.025 cm d^−1^, *b* is the soil cover fraction approximated as $b=\frac{LAI}{3}$ and $P_{tot}$ (mm d^−1^) is the total precipitation including above canopy irrigation on day *i*. LAI is derived from global average crop specific values (Breuer et al., 2003; Scurlock et al., 2001), which are reached after a linear increase from 0 m^2^/m^2^ to 0.1 m^2^/m^2^ over the initial and a further linear increase to the maximum average LAI over the developing growth stage similar to the crop coefficient (Fig. 2, Table 2). If above canopy irrigation (e.g. sprinkler irrigation) and precipitation occur on the same day, interception is attributed based on their relative fractions.

(7) $$Int_{P,i}=Int_{P+I,i}\cdot \frac{P_{i}}{P_{tot,i}}$$

(8) $$Int_{I,i}=Int_{P+I,i}\cdot \frac{I_{i}}{P_{tot,i}}$$

**Table 2:**
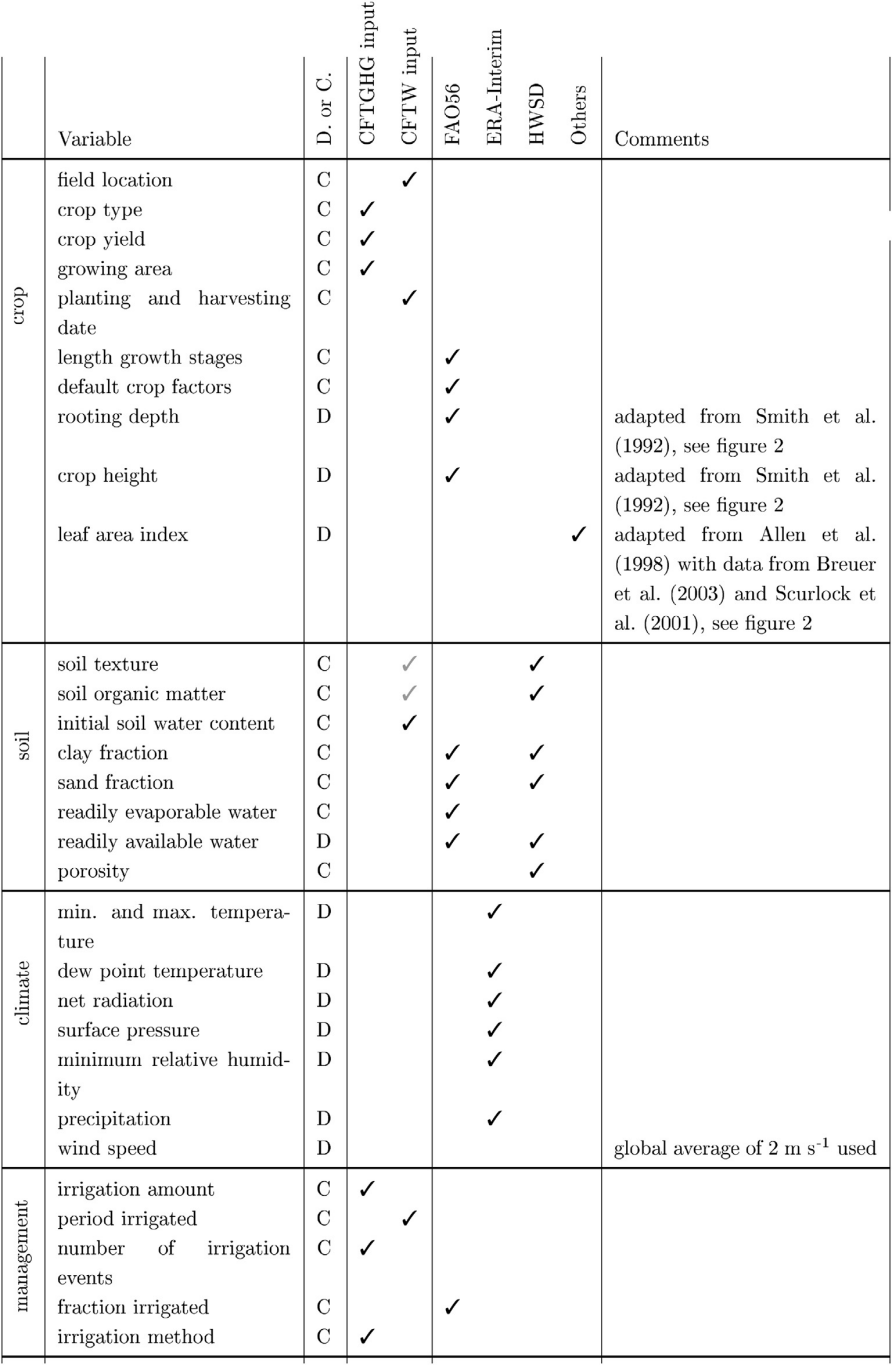
Data requirements for the CFTW and data-sources. ding51 indicates that data input is only optional. ding51shows that data input is mandatory. The column D. or C. indicates if the parameter is a constant (C) for the entire season or varies daily (D). CFTGHG input and CFTW input shows if the variable is new to CFT for the water module or has been part of the GHG model already.

Runoff $RO_{i}$ is determined using the approach of Jägermeyr et al. (2015) for the global dynamic vegetation model LPJmL.

(9) $$RO_{i}=1-P_{tot,i}\cdot \sqrt{1-\frac{\theta_{i}}{\theta_{SAT}-\theta_{WP}}}$$

where $P_{tot}$ refers to the sum of precipitation and all irrigation reaching the soil surface, *θ* is soil water content, $\theta_{WP}$ is soil water content at the permanent wilting point and $\theta_{SAT}$ is soil water content at saturation. After larger rain or irrigation events, soil water content may exceed field capacity and therefore water holding capacity of the soil and trigger deep percolation DP. FAO56 and CFTW work with the simplified assumption that all excess water above $\theta_{FC}$ drains into deeper soil layers the same day (Allen et al., 1998).

#### 3.1.3 Effect of management practices

The crop water use is controlled by many factors some of which cannot be altered nor managed by the farmer. Soil texture and climate including precipitation are defined by the field location. However, water usage is in some respects influenced by the farmer and these are reflected in CFTW.

First and foremost the choice of crop has a big influence on total $ET_{a}$. User can select 24 different crops, which vary with respect to growing period and length, $K_{c}$, stress tolerance (e.g. via RAW), crop height, rooting depth as well as LAI.

Organic matter content in the soil is important for determining the total water holding capacity and can be influenced by the crop producer for e.g. by reduced tillage or applying organic mulch as described in Cannell and Hawes (1994) and Mulumba and Lal (2008), respectively. This is implemented in CFTW by using the pedo-transfer function of Saxton and Rawls (2006), where field capacity and permanent wilting point is determined based on sand and clay content as well as soil organic matter. A higher organic matter content thus may reduce DP and increase resilience against water stress.

More options to impact $ET_{a}$ arise when irrigation is applied to the field. The model considers four different methods for irrigating: pivot, rain gun, flooding and drip irrigation. The methods vary with respect to their application efficiency as the model considers interception loss and runoff, with only infiltrating water being utilized by the crop. Irrigation also affects the initial crop factor $K_{c,ini}$ in two ways; firstly, $K_{c,ini}$ is determined by wetting interval as evaporation requires frequent wetting and, secondly, different irrigation practices wet different soil fractions (Allen et al., 1998). A smaller irrigated soil fraction, as for example when applying drip irrigation, where only 35% is wetted, implies lower evaporation. The wetted soil fraction for flood, pivot and rain gun irrigation, on the other hand, is 100% (Allen et al., 1998).

#### 3.1.4 Model outputs

The model determines the components of the soil water balance as discussed above. These results are used to estimate the crop irrigation requirements $I_{req}$ as follows:

(10) $$I_{req}=\sum \left(ET_{0}\cdot K_{c}\right)-\sum P_{net}$$

$P_{net}$ is the sum of net precipitation and net irrigation.

The tool provides the green and blue WFP (Hoekstra et al., 2011). The green water footprint $WFP_{green}$ reflects the total precipitation water used for the production of a crop, whereas the blue water footprint $WFP_{blue}$ reflects the used surface and groundwater via irrigation. Both WFPs are determined in accordance with the water footprint network as follows (Hoekstra et al., 2011):

(11) $$WFP_{blue}=\frac{min\left(I_{req},I_{net}\right)}{Y}$$

where $I_{net}$ is the part of the applied irrigation not lost via interception, surface runoff or deep percolation. The model does not consider losses related to water transport (conveyance efficiency). *Y* is the harvested yield in kg ha^−1^.

(12) $$WFP_{green}=\frac{\sum ET_{a}}{Y}-WFP_{blue}$$

$\sum ET_{a}$ describes the cumulative $ET_{a}$ of the entire growing season in l. The quotient of $\sum ET_{a}$ and *Y* is the total WFP of a crop. Water stored in the final harvested product is neglected because this generally consists of less than 1% of the total WFP and, in fact, is commonly in the order of 0.1% (Hoekstra et al., 2011).

### 3.2 Data

Table 2 and Fig. 1 provide an overview of data requirements. All data which is not required from the user is stored in a PostgreSQL database. The datasets include the Harmonized World Soil Database (HWSD), the ERA-Interim climate data, the FAO56 crop and soil parameters as well as a dataset of crop specific leaf area index (LAI) values.

ERA-Interim is a climate reanalysis dataset developed by the European Centre for Medium-Range Weather Forecasts (ECMWF) and it provides precipitation and meteorological variables for determining reference $ET_{0}$ and $ET_{a}$ according to the FAO56 and as described in the previous section (Dee et al., 2011). The three-hourly values available in the ECMWF database were adjusted for time zone and aggregated to daily values. The database provides climate data since the year 2004 and is updated every three months.

HWSD is the assimilation of multiple national and multinational soil databases (FAO, 2012) and is used to determine soil texture defined by sand, silt and clay content and organic mater content if the user does not provide this information. The pedo-transfer function of Saxton and Rawls (2006) are used to estimate field capacity and permanent wilting point based on this information.

The model includes crop factors, length of growing stages and other crop parameters for 25 different annual crops as well as perennial grass (See crop section in Table 2). Default values can be derived from Allen et al. (1998). Average LAI values are primarily based on two publications by Breuer et al. (2003) and Scurlock et al. (2001).

### 3.3 User interface

**Fig. 3:**
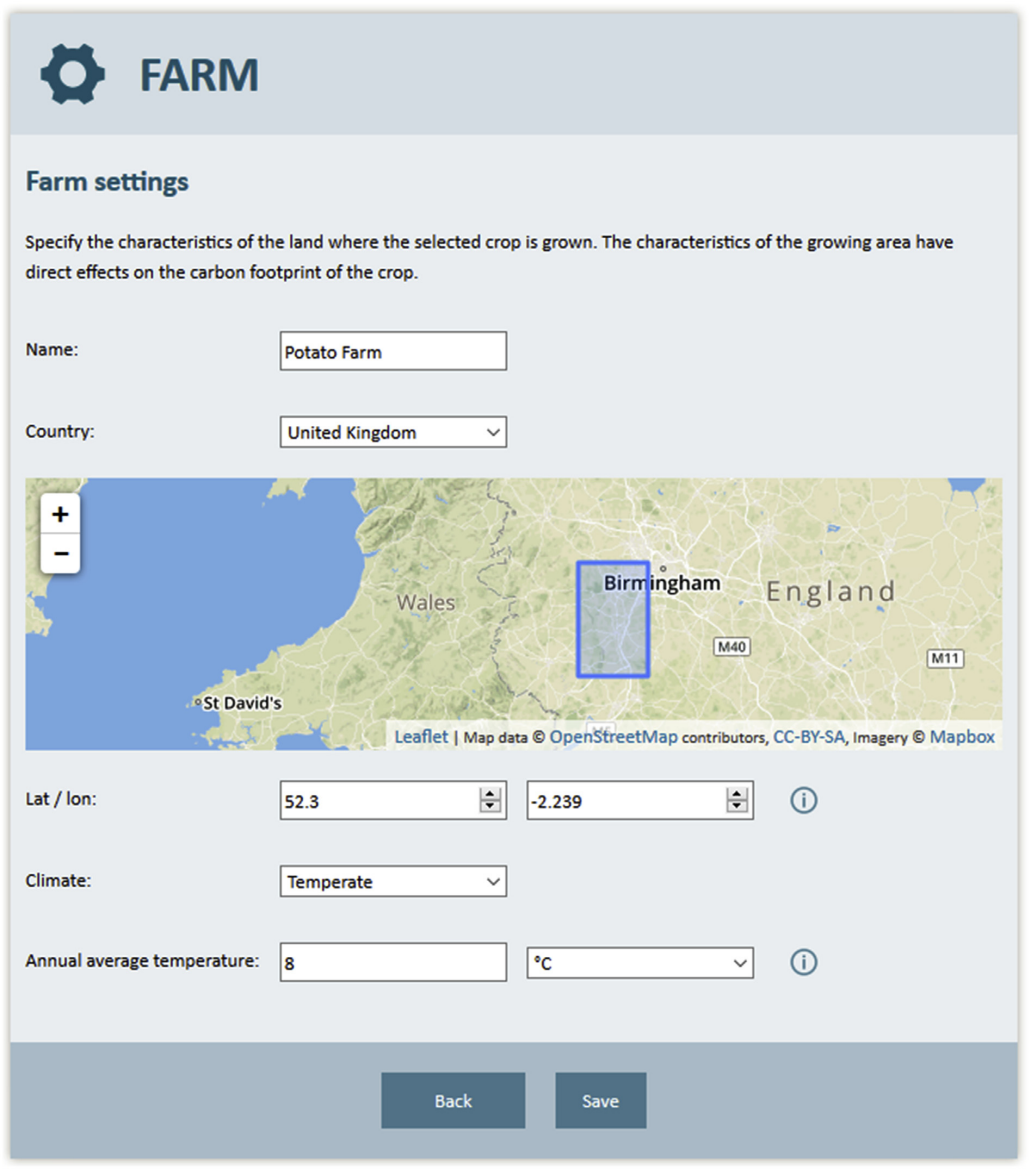

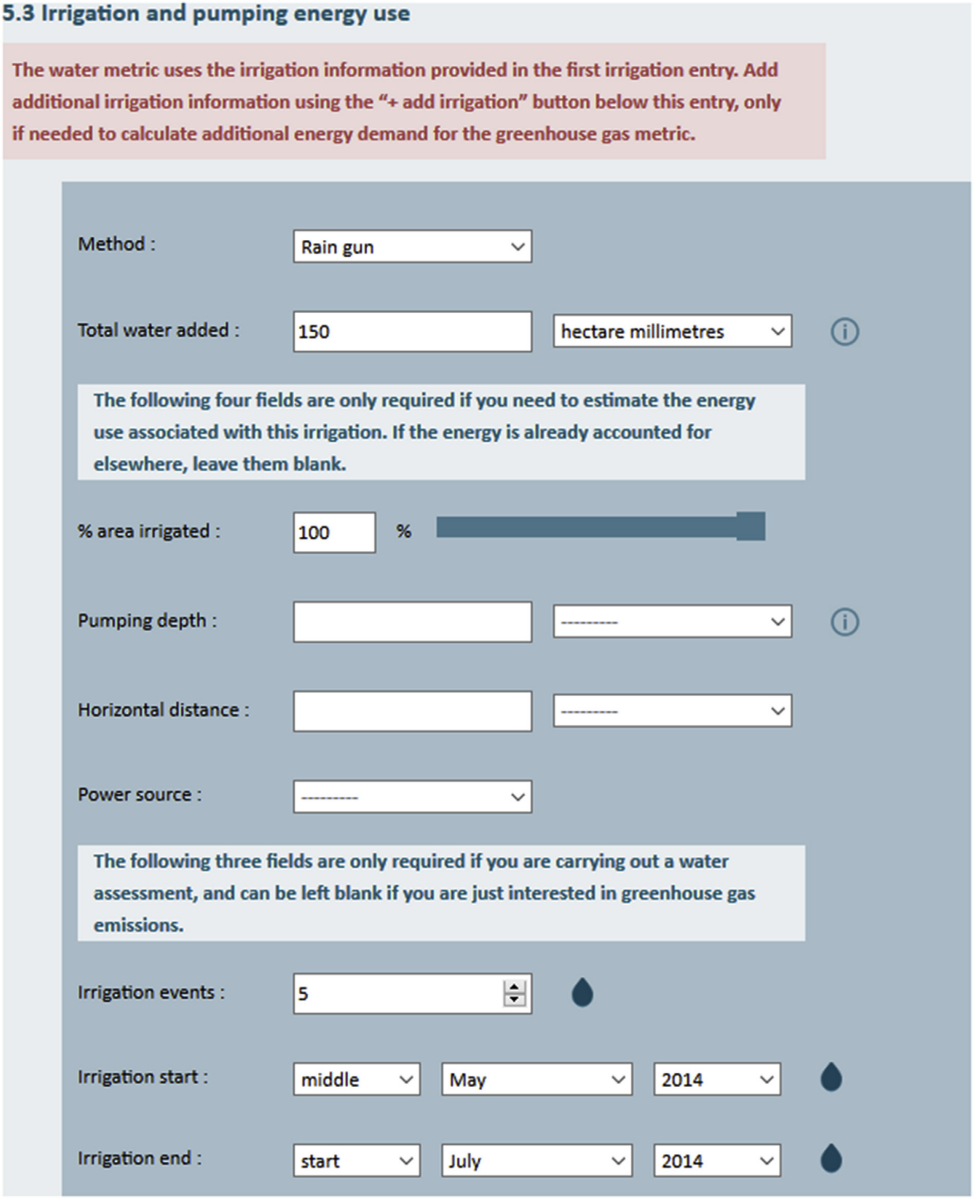
CFTW on-line input user interface. The figures show part of the user interface for a potato crop grown in England in 2014, which is irrigated between May and early July using a rain-gun system.

**Fig. 4:**
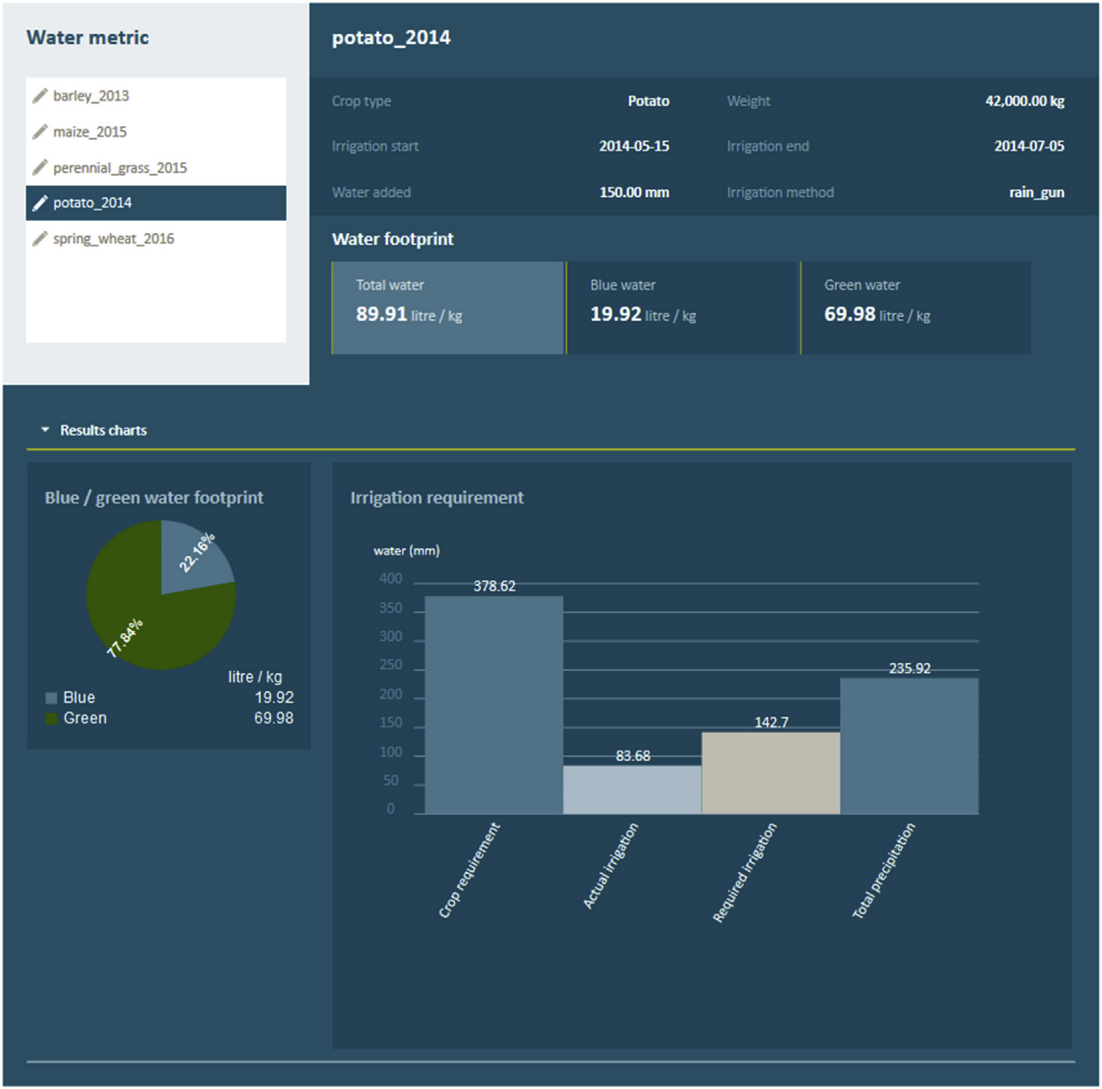
CFTW on-line results for a potato crop in England as described in Fig. 3.

The CFTW user interface is fully integrated into the CFT to avoid redundancies of input variables between the GHG calculator and the water tool. For example, some inputs, such as crop and growing area, are required for both metrics of the CFT. Several questions presented to the user, such as intensity and average temperature are, however, only relevant for the GHG metrics and have no influence on the water results (see Fig. 3). The input and output user interfaces are designed to make water assessments easily accessible via an interface that is quick and self-explanatory as displayed in Fig. 3, Fig. 4. All relevant user inputs for the water component are presented in Table 2.

Input fields required only for CFTW concern field location, growing period, initial soil moisture and irrigation management and are highlighted using a droplet icon. For all inputs which require a unit the on-line tool provides a selections of units from which to choose. The location is entered via “Farm settings”, where the user can select the field location by providing longitude and latitude or by tagging the location on a map (Fig. 3). Growing and irrigation period are not provided as dates, but as early, middle or late in a given month and year representing the 5th, 15th and 25th day of each month respectively. The total irrigation amount is distributed equally between both dates and the total number of irrigation events. Initial soil moisture content is a required input and can be entered as high (soil moisture at field capacity), mid (2/3 of available water capacity filled) and low (1/3 of available water capacity filled). The use of approximate dates and classes were identified to be a suitable compromise between the accuracy in the input and model usability since specific values are not always available.

The blue and green WFP and the irrigation requirements based on the assessment of the entire growing period are displayed in the results section (see Fig. 4). Furthermore, additional information about the results are provided after pressing the info icons on the results page.

## 4 Assessment of CFTW

The assessment of CFTW was done in three ways: First we compare the CFTW estimates with field observations, which represents a very time consuming, but reliable approach to assess total water footprints. This is done by using different field trials of water footprints in the scientific literature as well as different eddy covariance measurement sites (see Appendix A). Secondly, model results are compared with available estimates by the water footprint network (WFN), which are quickly accessible and represent state level averages. In a final step we analyse the usability of the tool based on feedback we have received by members of the CFA.

The goal of this study is not to analyse the quality of each input dataset individually, but to provide a functional evaluation of the model results based on all input data used and test if CFTW responds to differences in management, climate, soil and crop.

### 4.1 Experimental tests

#### 4.1.1 Case studies

CFTW was tested using observations from 16 published crop water productivity studies for potato, wheat and maize as those represent the most commonly used crops in CFT. Each of the studies explored between 1 and 18 different trials. The studies were selected to represent different irrigation management practices, climates, soils and potential yields in order to investigate the response to these important drivers. The selected studies are presented in Table 3. The evaluation is only based on studies that use site observations as soil water balance measurements, lysimeter studies or eddy covariance measurements. Modelling studies were not used in order to avoid interdependencies in modelling results. Model runs were performed using all available information about growing period, irrigation design and soil. In contrast to the on-line model interface, this study uses exact dates for sowing and harvesting as well as beginning and end of irrigation. Furthermore, the trials also include a fifth irrigation method representing furrow irrigation with a soil wetting fraction of 50% which is not yet available on-line.

**Table 3:** The table provides information on the 16 studies used for validating CFTW. Most studies are using the soil water balance method (SWB) to determine $ET_{a}$. Other methods used are lysimter studies (LM), eddy covariance measurements (EC) and the Bowen ratio (BR).

Study	crop	location	country	method	study aim (assessing impact of)
Aksic et al. (2014)	potato	43.3 N, 21.9 E	Serbia	SWB	irrigation amount
Ati et al. (2012)	potato	33.3 N, 44.2 E	Iraq	SWB	irrigation method & amount, fertilizer rates
Bandyopadhyay and Mallick (2003)	wheat	23.0 N, 88.1 E	India	SWB	irrigation amount
Corbeels et al. (1998)	wheat	33.9 N, 5.6 W	Morocco	SWB	irrigation amount, fertilizer rates
Cossani et al. (2012)	wheat	41.2 N, 1.1 E	Spain	SWB	irrigation amount, fertilizer rates
Erdem et al. (2006)	potato	41.0 N, 27.5 E	Turkey	SWB	irrigation method, amount & interval
Fengrui et al. (2000)	potato, maize	35.7 N, 107.9 E	China	SWB	crop rotations
Hernández et al. (2015)	maize	37.8 S, 58.3 W	Argentina	SWB & Micro-LM	irrigation amount, fertilizer rates
Igbadun et al. (2008)	maize	8.6 S, 33.9 E	Tanzania	SWB	irrigation interval & periode
Jabro et al. (2012)	potato	48.2 N, 103.1 W	USA	SWB	irrigation interval
Jia et al. (2014)	wheat	36.2 N, 117.2 E	China	SWB	irrigation amount & method
Kang et al. (2000)	maize	38.0 N, 103.1 E	China	SWB	irrigation amount & method
López-Urrea et al. (2009)	wheat	39.2 N, 2.1 W	Spain	LM	- (only single wheat crop)
Parent and Anctil (2012)	potato	46.8 N, 72.3 W	Canada	EC	- (only single potato crop)
Suyker and Verma (2009)	maize	41.2 N, 96.5 W	USA	EC	irrigation amount
Young et al. (2008)	wheat	31.7 S, 150.5 E	Australia	SWB & BR	–

Furthermore, state level WFP data published by the WFN and CFTW WFPs are compared to the observed total WFPs of the case studies. The WFN values are part of a global modelling study for various crops using a grid based water balance model also derived from Allen et al. (1998) using global datasets for crop distribution, precipitation, long-term monthly $ET_{0}$ and soil properties (Mekonnen and Hoekstra, 2011). Soil and climate data used for WFN estimates differ from datasets used for CFTW and are further described in Mekonnen and Hoekstra (2011). Results have been aggregated for administrative units and results are representative for the years 1996 until 2005 (Mekonnen and Hoekstra, 2011). The comparison highlights the potential differences between state level averages and local agricultural practices for a specific year.

#### 4.1.2 Result and discussion of case studies

**Fig. 5:**
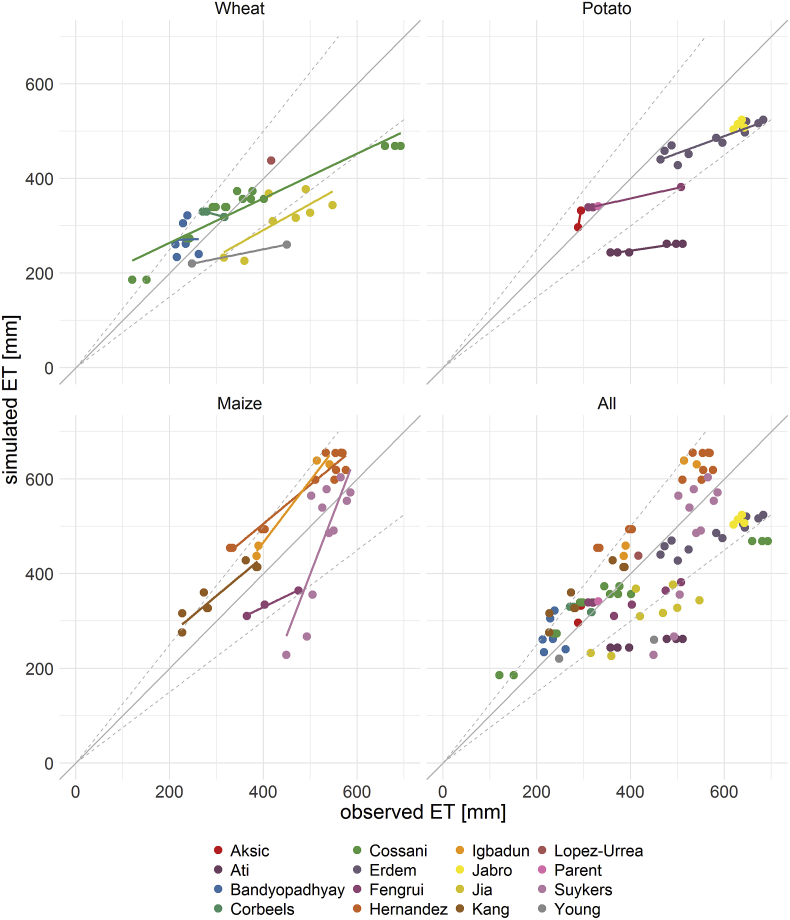
Simulated $ET_{a}$ using CFTW versus observed $ET_{a}$ for 16 different studies for wheat, potato and maize. The dashed lines indicate an offset between simulated and observed $ET_{a}$ of more than 25%.

CFTW explained more then 50% of all the variance in all 16 studies of observed $ET_{a}$ ($R^{2}$  = 0.53, p-value < 0.05). The best results are obtained for potato ($R^{2}$  = 0.63, p-value < 0.05), followed by wheat ($R^{2}$  = 0.61, p-value < 0.05) and maize ($R^{2}$  = 0.57, p-value < 0.05). The Root Mean Square Error (RMSE) of all studies combined is 103 mm and ranges between 28 mm for Aksic et al. (2014) and 190 mm for Ati et al. (2012) (Fig. 5).

The median relative error between simulated and observed $ET_{a}$ is 1.3% with an inter-quartile range of −20.2% and 15.5% and thus shows no clear bias towards over- or underestimation of $ET_{a}$. The model reproduced a significant positive correlation for 10 out of 13 studies with more than two trials (Fig. 5). Only results for Corbeels et al. (1998) show a significant negative correlation. This shows that, based on 13 studies, CFTW correctly identifies water management improvements. The magnitude of change in $ET_{a}$ is however underestimated for 8 and overestimated for 2 of the 10 studies - with significant positive correlation. In cases where underestimation of the change in $ET_{a}$ occurs, this may in fact also result from the method of measurement of $ET_{a}$. Most studies used in this work are based on soil water balances which tend to underestimate $ET_{a}$, in particular for high precipitation or irrigation (Sadras and Angus, 2006) due to the fact that these studies often neglect runoff and deep percolation.

The biggest positive relative error between model results and measurements is from a trial of Cossani et al. (2012). $ET_{a}$ is overestimated by over 54.4% and 65.4 mm. The trial shows a control trial of the study without any irrigation and only little precipitation, which means $ET_{a}$ is highly sensitive to initial soil moisture. CFTW currently permits only three levels of initial soil moisture with the lowest being one-third of the available water capacity. This may therefore lead to an overestimation of the available water in the soil, when actual soil moisture is below this at the time of sowing as in this trial.

The greatest underestimations occurred at individual trials from Suyker and Verma (2009), Ati et al. (2012), Young et al. (2008) and Jia et al. (2014). Suyker and Verma (2009) shows a substantial underestimation of precipitation in ERA-Interim during summer months in 2003 and 2005, which triggers water stress and an underestimation of rain-fed trials. Other reasons may also contribute to the discrepancies, since rainfall is not underestimated in 2001 while simulated $ET_{a}$ is 150 mm lower than observed. Nevertheless, the rain-fed trial in 2001 shows the smallest error of all trials without irrigation in Suyker and Verma (2009).

Data points from Ati et al. (2012) represent furrow and drip irrigated potato grown between September and January with different fertilizer application levels. $ET_{a}$ from all trials in this study are underestimated by CFTW. The model results do not show crop water stress and therefore indicates that $ET_{c}$ is underestimated. $ET_{0}$ is high in the beginning, but decreases towards the end of the growing season when crop factors are higher. Therefore, the underestimation is because $ET_{0}$ is underestimated during the winter months or the $K_{c}$ in the early month of the growing season is too low, possibly linked to an underestimation of soil wetting fraction. In addition, different levels of fertilization - and thus different crop growth curves per crop - are currently not implemented in CFTW which assumes optimal nutrient levels for all crops. Hence the model results show no variance in $ET_{a}$ associated with different fertilization rates. The strong offset of the Ati et al. (2012) $ET_{a}$ estimates results in a mean relative error of −17% for all potato studies.

For Jia et al. (2014) the reasons are more complex and the underestimation of $ET_{a}$ cannot be clearly attributed. The initial crop factor is very low, which leads to a low water use in the beginning, which again triggers great deep-percolation. Moreover, the results show high crop water stress in April and June at the end of the growing season.

**Fig. 6:**
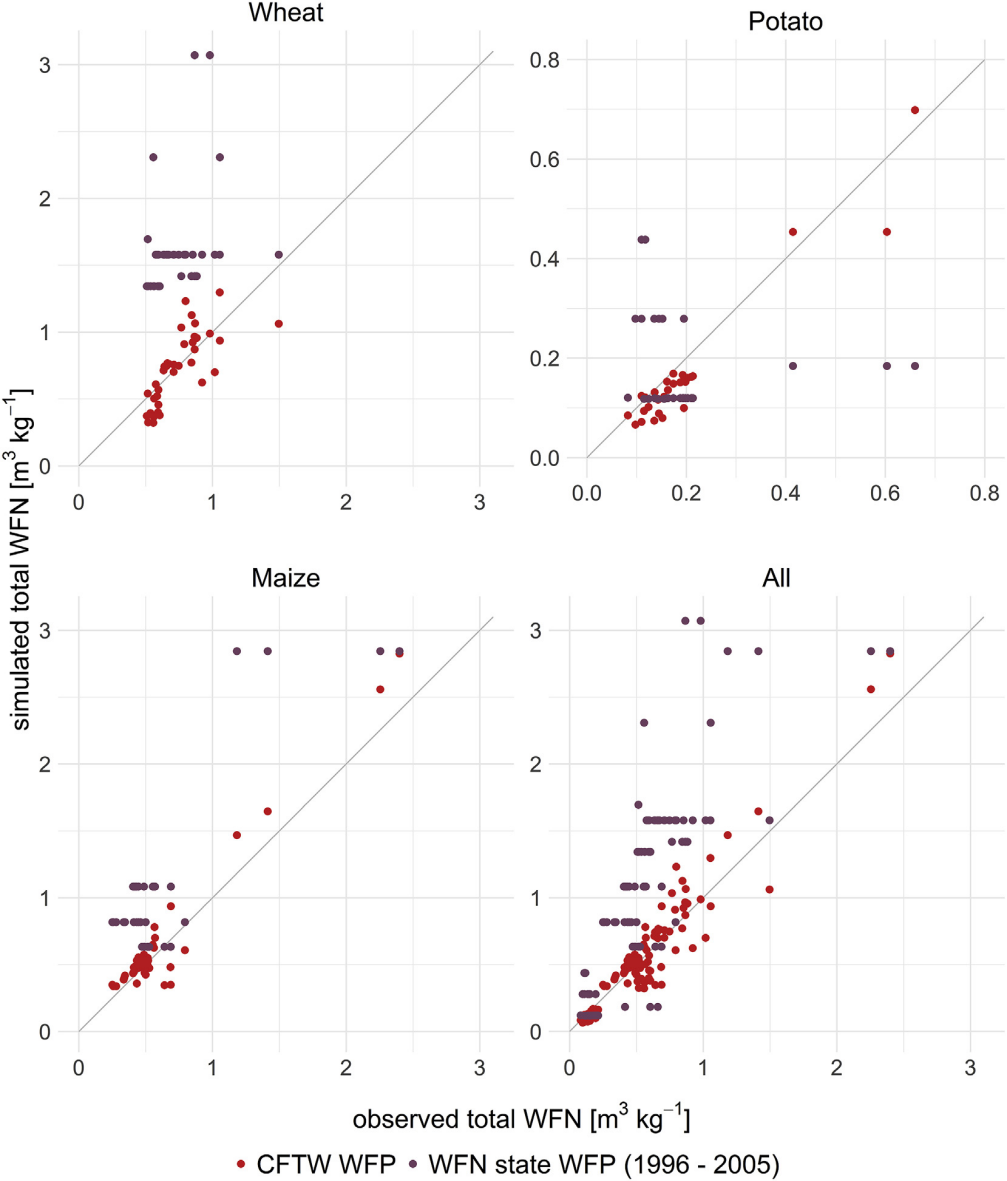
Comparison of state level total WFP estimates by WFN for 1996 to 2005 and CFTW total WFPs to observed total WFPs of the 16 case studies. Two points are removed from the wheat plot where observed water footprints exceed 3.0 m^3^ kg^−1^, to enhance visibility of the remaining studies.

The observed total WFPs of all studies range between 0.083 m^3^ kg^−1^ and 8.686 m^3^ kg^−1^ (Fig. 6). The WFPs above 1.500 m^3^ kg^−1^ belong to trials with little irrigation or precipitation causing low yields and thus often served as control trials. Only four trials of Corbeels et al. (1998) and Igbadun et al. (2008) exceed a total WFP of over 1.500 m^3^ kg^−1^. The underestimation of potato $ET_{a}$ also leads to an underestimation of potato water footprints. Nevertheless, CFTW accounts for 92.6%–99.1% of the variance of all WFPs for the specific crop (potato: $R^{2}$  = 0.926, p-value < 0.05; wheat: $R^{2}$  = 0.991, p-value < 0.05).

In contrast, WFPs estimated based on the WFN state level are much more diverse. Using CFTW with limited user input and global datasets reduces the RMSE of the WFP by over 70% for all crops in comparison of WFN estimates (Table 4, Fig. 6). This shows the differences between local WFPs and average state level WFPs presented by WFN, but also the benefit of using field level yield data as well as more local climate, soil and management information. Therefore, WFN state level WFP data cannot be used as approximation for individual fields within one state as it does not reveal the variability of WFPs on state and even individual field level.

**Table 4:** The table displays the RMSE for estimated WFPs using CFTW or WFN state level values representative for 1996 to 2005.

Crop	RMSE for WFN WFP [m^3^ kg^−1^]	RMSE for CFTW [m^3^ kg^−1^]
potato	0.174	0.048
wheat	1.442	0.404
maize	0.555	0.154
all	0.941	0.264

### 4.2 Usability trials

The tool was tested with users via presentations to candidate user groups in the form of a webinar, a workshop and individual trials organized by the CFA between February and June 2017. Here the importance of science based methods for voluntary assessments in the agricultural and cooperate sector was emphasized in order to assess, improve and communicate the sustainability of crop production as well as global supply chains. During these trials, it was recognized how CFTW has provided a practical tool for the assessment of agricultural water use and increased the usability of FAO56 by, (i), limiting the user input to basic questions that constitute common farmer knowledge and, (ii), integrating a climate, soil and crop database. Users, however, acknowledged that the use of gridded climate data, default crop parameters and soil data may not capture the spatial or crop specific variability in those domains (see Appendix A). Therefore, results should be interpreted with caution when these values do not well represent local conditions. In addition, the tool tried to minimize user input by integrating the GHG and water user interface. While this reduced the redundancy of questions in CFT significantly, it may also lead to questions when the input is not explicitly allocated to the GHG or water metrics. Therefore, defining user pathways depending on user interest is desirable and should be considered in further developments.

CFTW enables users to assess their green and blue WFP considering local meteorology, soil and harvest, capturing different growing seasons and the annual variability of the weather. Although Hoekstra et al. (2011) also addresses farmers and gives recommendations on how to reduce the WFP of crop production, uptake by farmers has been low, whereas a focus on irrigation requirements, water productivity and crop water stress appears to have greater meaning with farmers.

The discussion with members of the CFA also revealed difference of opinion and uncertainty on how to best assess the environmental impact of water consumption and how to define reduction targets. While some showed a strong interest in the WFP, others target a reduction of abstracted blue water, an increase in irrigation efficiency or avoiding water scarcity and risk. The discussion observed here is also present in the scientific literature where recommendations vary to the point of the reduction of the WFP to an approach that focuses on water scarcity (Boulay et al., 2015; Hoekstra et al., 2011; ISO, 2014; Ridoutt and Pfister, 2010).

### 4.3 Limitations and possible improvements

In this study and in trialling of the tool we have identified several specific areas where there is scope for further development and opportunities for improvement. This could mean an improvement of default data provided in the tool, enhancing the model itself or an advancement of the user interface.

Pereira et al. (2015) and Allen et al. (1998) emphasize the importance of accurate measurements of meteorological variables to reduce the uncertainty for $ET_{0}$. CFTW uses global gridded data to determine $ET_{a}$ based on FAO56 and is therefore taking a similar approach as Siebert and Döll (2010) and Mekonnen and Hoekstra (2011). The average climate in the grid cell may not represent the meteorology at the field location for various reasons, as for example, topography. In particular ERA Interim precipitation data is linked to uncertainties due to the spatial variability of rainfall. Furthermore, some studies show an underestimation of ERA Interim precipitation (de Leeuw et al., 2015; Szczypta et al., 2011), similar to what was observed for the trials of Suyker and Verma (2009). Still, a refined analysis of using local meteorological data versus ERA Interim data for CFTW using 10 eddy covariance measurement sites show only a small improvement when using local data (see Appendix A). Furthermore, the outline of the 0.75^°^ grid cell that is used under “Farm settings” enables the user to assess how representative weather is across this area based on own local knowledge. Still, future versions of the model may consider using meteorological data with a higher spatial resolutions, such as the newly released ERA5 dataset (ECMWF, 2018) or allow for the replacement of individual climate variables with local meteorological data if available.

The tool might also make better use of the data available in the HWSD, by displaying the different soil textures available in the HWSD for a given location and offering the user to select the most representative soil from these options rather than defaulting to the most abundant soil texture as is done currently. Moreover, the quality of the HWSD varies strongly across different world regions and countries (Avellan et al., 2012; FAO, 2012). It is possible that alternatives such as the newly available high resolution soil map SOLIDGRIDS could replace HWSD in the future (Hengl et al., 2017) and address this known issue. Nevertheless, the use of local soil data whenever available is likely to be the most reliable option.

Furthermore, default crop coefficients, rooting depth, crop height and LAI are currently set internally and not by the user. Since global averages may not be representative at farm level (Allen et al., 1998), we propose to improve the on-line tool in the future to enable users to overwrite this crop data where desirable. Still, CFTW is different to most other models as it determines initial $K_{c}$ based on wetting frequency and soil texture and adjusts the remaining $K_{c}$s automatically and therefore eliminates a great source of error present in most tools (Pereira et al., 2015).

The model itself can be improved by enhancing current model components or increasing its scope. For example, the water balance estimated in CFTW is similar to CROPWAT (Smith, 1992) and does not account for any impermeable layers or capillary rise from groundwater layers. Raes et al. (2012) have overcome this limitation in AquaCrop by enhancing the input requirements for the tool. In addition, the pedo-transfer function used for determining water holding capacity for the soil profile in CFTW was calibrated for top soils and is applied here for the full rooting depth. This could be replaced by a different pedo-transfer function to reduce uncertainties for water holding capacity.

CFTW represents water stress and assumes that crop growth is not limited by other factors as nutrients, temperature or salt stress. Inclusion of these features could increase the scope of the tool as has also been done in AquaCrop (Raes et al., 2012). In addition, more management interventions such as mulching, contour ploughing, fertilizing or further irrigation methods to reduce runoff and decrease evaporation as well as transpiration might be included. Future developments can show the benefits of such practices on water management and therefore would give greater relevance to the tool and encourage adoption and reporting of these practices. Mulching, for example, is estimated to reduce soil evaporation by 50% per area covered (Allen et al., 1998; Chukalla et al., 2015).

Currently the tool only reflects the water use element of the WFP estimation while yield is defined by the user. Coupling CFTW with a crop growth model could help to show the co-benefits of different management for water and yield and therefore show the full reduction potential of the WFP.

Finally, the WFP has been criticised in the past for not being easily comparable and not reflecting local water scarcity (Ridoutt and Pfister, 2010). In practice it is more important to manage water efficiently when a river basin suffers from water scarcity and the WFP does not convey the importance of this context. The tool (and any reporting of WFP) would therefore benefit from provision of information on local water scarcity or availability.

These examples show how existing model components can be further developed in the future. Most of the changes discussed above, would imply an adjustment to the user interface and higher user input. Thus, all changes have to be thoroughly evaluated based on the added accuracy and functionality, while remaining a user friendly tool, which is easy to use. This can only be done and decided in close collaboration with targeted user groups, as conducted so far. This also holds true for more precise user input for already existing input parameters, as for example, irrigation scheduling and initial soil moisture.

While we show that CFTW provides reliable estimates based on 16 field studies, further testing is essential to consider a wider range of management interventions, climates, crops and soils. The estimation and communication of uncertainties within the tool remains an important task in terms of model evaluation and usability. Using various environmental conditions and management during testing showed that CFTW is sensitive to those changes and that ERA Interim is sufficiently accurate. A sensitivity assessment of CFTW considering the uncertainties for crop, soil and climate input using information from additional field sites is foreseen.

## 5 Conclusion

The CFTW is to our knowledge the first on-line water tool for farmers, suppliers, NGOs and consumer goods producers that provides WFP results and irrigation requirements using gridded climate data, global soil maps and local management information. It overcomes some of the main constraints with current models as it provides default input data where users find provision of such data difficult, uses terminology known to the farmer and has an on-line user interface. The strong collaboration with the Cool Farm Alliance helped us to shape the tool based on demand and enabled us to make scientific models and datasets available to end-users.

The validation of CFTW using 16 studies for potato, wheat and maize in 12 different countries with a total of 106 observations showed that the CFTW was effective in modelling $ET_{a}$ and total WFP and is able to indicate the correct direction of change in water use for management interventions or location changes for most studies investigated.

In contrast, the long-term and spatially averaged results provided by the WFN were not able to represent local conditions. By that, it is shown how CFTW helps crop producers to identify adaptation strategies relevant for the specific local conditions. Finally, by integrating this water assessment tool with the already existing on-line CFT developed for the assessment of green-house gases emissions (Hillier et al., 2011) and the biodiversity module (CFA, 2016), it provides a unique platform to engage farmers and users towards a holistic assessment of the agricultural sector.

## Figures and Tables

**Table A1:** Eddy covariance sites from AmeriFlux and European Carbon Flux that were used for testing model performance based on climate input data. Sites were used for minimum and maximum temperature, precipitation, net radiation, surface pressure and wind speed to drive CFTW.

site	crops	years	location	country	reference
CH-Oe2	Wheat, Potato, Barley	2005–2007	47.3N, 7.7E	Switzerland	Dietiker et al. (2010) and Emmel et al. (2018)
DE-Kli	Maize, Barley, Maize	2007, 2008, 2011, 2012	50.9N, 13.5E	Germany	Prescher et al. (2010)
FR-Gri	Maize, Barley, Wheat, Triticale	2005–2011	48.8N, 2.0E	France	Loubet et al. (2011)
IT-BCi	Maize	2004, 2005	40.5N, 15.0E	Italy	Vitale et al. (2007, 2009)
US-ARM	Maize	2004–2006, 2009	36.6N, 97.5W	USA	Fischer et al. (2007)
US-Bo1	Maize, Soybean	2001–2006	40.0N, 88.3W	USA	Meyers and Hollinger (2004) and Wilson and Meyers (2007)
US-IB1	Maize, Soybean	2006, 2008	41.9N, 88.2W	USA	Matamala et al. (2008)
USeNe1	Maize	2002–2012	41.2N, 96.5W	USA	Suyker and Verma (2009)
USeNe2	Maize, Soybean	2002, 2004–2012	41.2N, 96.5W	USA	Suyker and Verma (2009)
USeNe3	Maize, Soybean	2002, 2004–2012	41.2N, 96.4W	USA	Suyker and Verma (2009)

**Fig. A1:**
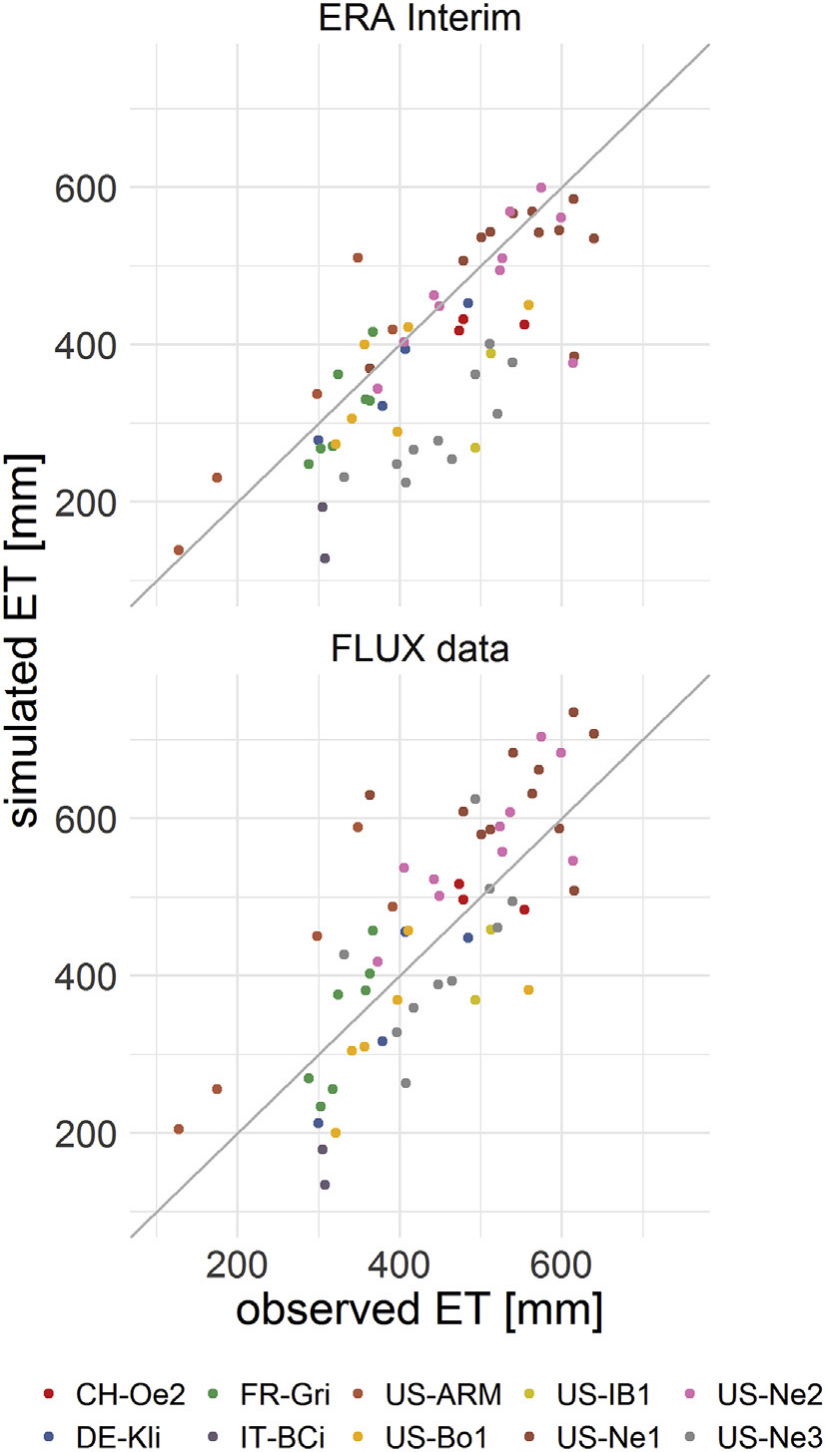
CFTW model results for $ET_{a}$ based on local (FLUX data) and global (ERA Interim) climate data input compared to observations based on eddy covariance measurements.

**Fig. A2:**
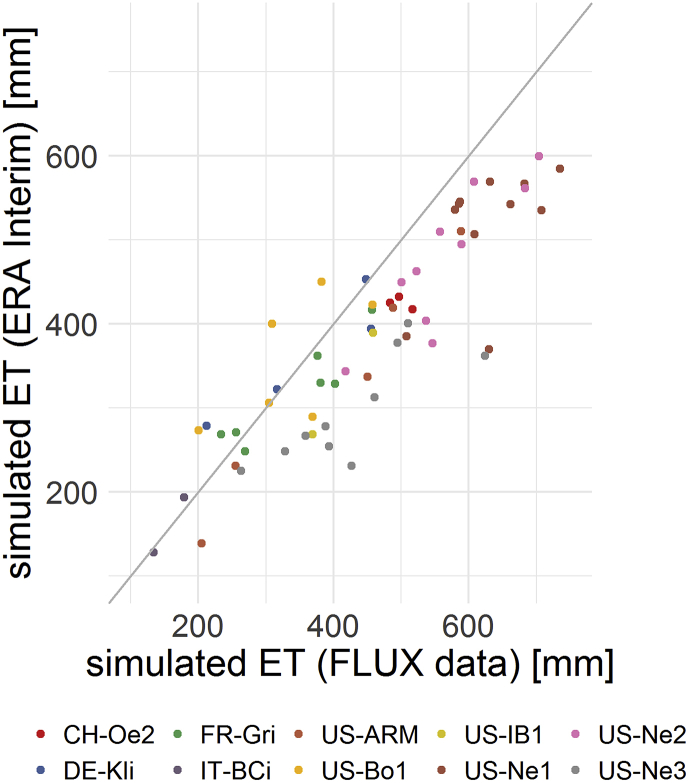
CFTW model results for $ET_{a}$ based on local (FLUX data) and global (ERA Interim) climate data input.

## Appendix

### A Testing model performance based on climate data input

CFTW was further tested for climate data uncertainty. The model is based on the global climate dataset ERA Interim, which has been evaluated independently in several studies (Gao, 2013; Thiemig et al., 2012; Zhang et al., 2016). This section provides an analysis of model results based on field data input at crop fields and ERA Interim input.

Field data was used from 10 eddy covariance measurement sites of the AmeriFlux network and European Fluxes Database Cluster using local information on daily maximum and minimum temperature, net radiation, wind speed, atmospheric pressure and precipitation if available (Table A1). This data was used as it provides field relevant meteorological data, agronomic information as well as observed $ET_{a}$. As field data contained gaps we allowed for an up to 10% gap filling using ERA Interim data, except for atmospheric pressure where we used ERA Interim if this was not observed at the eddy covariance site. Furthermore, CFTW required dewpoint temperature, which was not observed at any of the sites and therefore was replaced with daily minimum temperature as recommend by FAO56 (Allen et al., 1998). Observed $ET_{a}$ was gap filled using linear interpolation and only for a maximum of 5% of the growing season.

Our comparison based on 10 eddy covariance sites and a total of 60 individual observations indicated that CFTW performs only slightly better when using local meteorological input ($R^{2}$  = 0.59, RMSE = 97 mm) compared to ERA Interim input ($R^{2}$  = 0.53, RMSE = 100 mm) (Fig. A1). Furthermore, CFTW $ET_{a}$ results driven by local and global climate data showed a very good agreement ($R^{2}$  = 0.78) (Fig. A2). However, we also found that CFTW results based on ERA Interim were on average 12.5% lower compared to model runs based on local data. This was driven by 3 sites (US-Ne1, US-Ne2 and US-Ne3) located in the same ERA Interim grid cell, due to an average underestimation of precipitation of 41.3%–56.7% (see also 4.1). The bias was significantly reduced (−4.9%) when these sites were excluded from the analysis.

Therefore, we conclude that ERA Interim is sufficiently accurate for the purpose of CFTW, but may benefit from improved precipitation input.

## Footnotes

Appendix B Supplementary data to this article can be found online at https://doi.org/10.1016/j.jclepro.2018.09.160.
