# Supplementary material for: Cool Farm Tool Water: A global on-line tool to assess water use in crop production
Source: J Clean Prod. 2019 Jan 10;207:1163–79. doi: 10.1016/j.jclepro.2018.09.160 (PMC6771653; doi:10.1016/j.jclepro.2018.09.160)
Supplement: Supplementary file 1 [file mmc1.pdf]

Appendix

B. Schematic representation of CFTW model

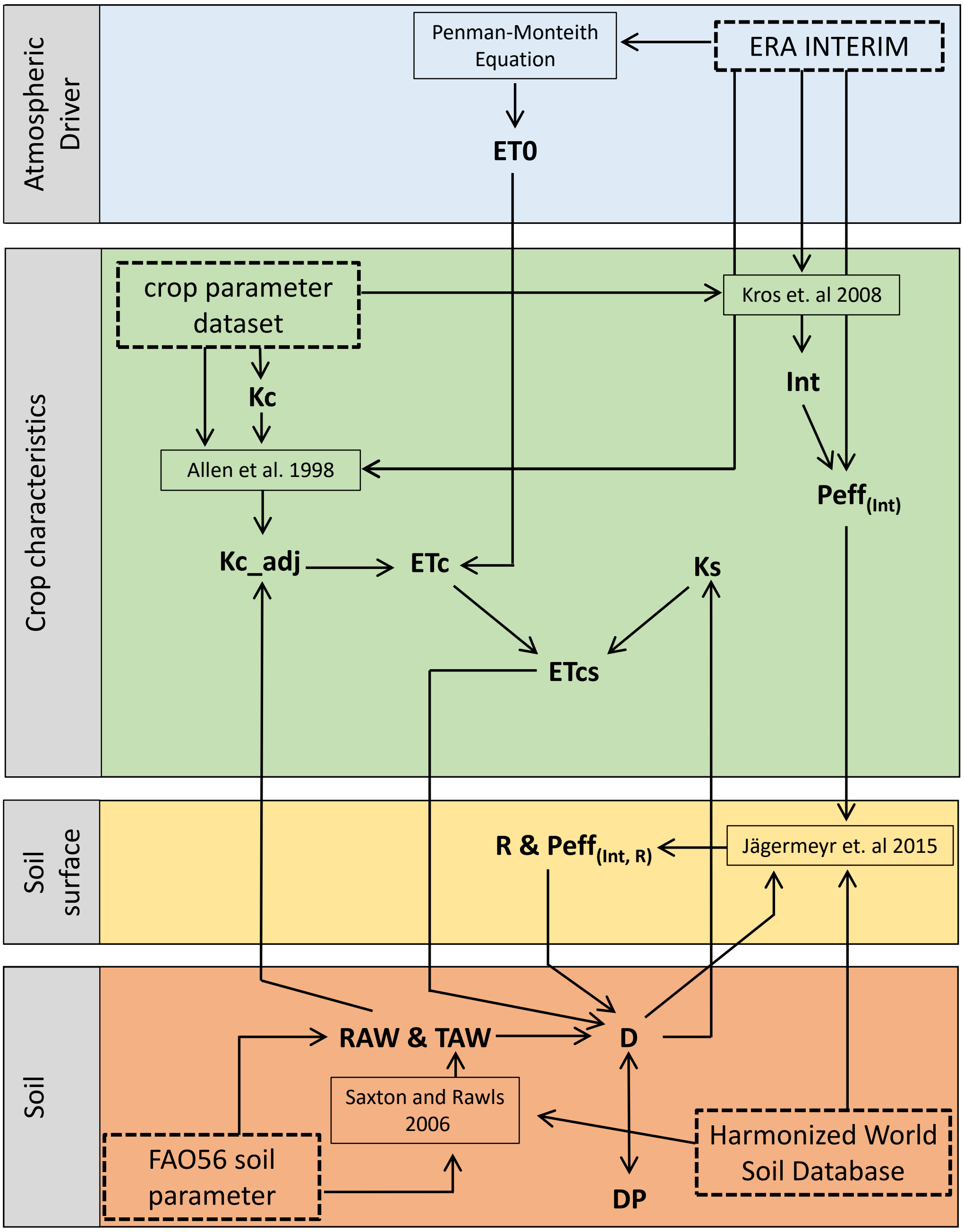

RAW: Readily available water, **TAW** : Total available water, **DP**: Deep percolation, **D**: Soil water depletion, **Int**: Interception, **Ks**: Crop water stress, **Kc_adj**: Adjusted crop growth curve, **Kc**: Crop growth curve, **ET0**: Reference evapo-transpiration, **R**: Runoff, **ETc**: Potential crop evapo-transpiration, $\mathbf{Peff}_{(\mathrm{Int})}$: Precipitation – interception, $\mathbf{Peff}_{(\mathrm{Int,\ R})}$: Precipitation - interception - runoff, **ETcs**: Crop evapo-transpiration including water stress

Figure B1: Schematic representation of CFTW model. The dashed boxes represent datasets used in the model and stored in the PostgreSQL database. The boxes represent sub-models or related publications employed in CFTW.
